# The development of growth hormone-releasing hormone analogs: Therapeutic advances in cancer, regenerative medicine, and metabolic disorders

**DOI:** 10.1007/s11154-024-09929-2

**Published:** 2024-11-26

**Authors:** Andrew V. Schally, Renzhi Cai, Xianyang Zhang, Wei Sha, Medhi Wangpaichitr

**Affiliations:** 1 Interdisciplinary Stem Cell Institute, University of Miami Miller School of Medicine, Miami, FL USA; 2 Miami VA Healthcare System, Endocrine and Polypeptide Institute, Miami, FL USA; 3 Department of Medicine, University of Miami Miller School of Medicine, Miami, FL USA; 4 Department of Pathology, University of Miami Miller School of Medicine, Miami, FL USA; 5 Sylvester Comprehensive Cancer Center, Miami, FL USA; 6 Department of Surgery, Division of Thoracic Surgery, University of Miami Miller School of Medicine, Miami, FL USA; 7 South FL VA Foundation for Research and Education, Miami, FL USA

**Keywords:** GHRH agonists, GHRH antagonists, GH release, Regenerative activity, Cancer therapies

## Abstract

Growth Hormone-Releasing Hormone (GHRH) and its analogs have gained significant attention for their therapeutic potential across various domains, including oncology, regenerative medicine, and metabolic disorders. Originally recognized for its role in regulating growth hormone (GH) secretion, GHRH has since been discovered to exert broader physiological effects beyond the pituitary gland, with GHRH receptors identified in multiple extrahypothalamic tissues, including tumor cells. This review explores the development of both GHRH agonists and antagonists, focusing on their mechanisms of action, therapeutic applications, and future potential. GHRH agonists have shown promise in promoting tissue regeneration, improving cardiac function, and enhancing islet survival in diabetes. Meanwhile, GHRH antagonists, particularly those in the MIA and AVR series, demonstrate potent antitumor activity by inhibiting cancer cell proliferation and downregulating growth factor pathways, while also exhibiting anti-inflammatory properties. Preclinical studies in models of lung, prostate, breast, and gastrointestinal cancers indicate that GHRH analogs could offer a novel therapeutic approach with minimal toxicity. Additionally, GHRH antagonists are being investigated for their potential in treating neurodegenerative diseases and inflammatory conditions. This review highlights the versatility of GHRH analogs as a promising class of therapeutic agents, poised to impact multiple fields of medicine.

## Introduction

Growth hormone-releasing hormone (GHRH) is a hypothalamic hypophysiotropic peptide that plays a central role in regulating the synthesis and secretion of growth hormone (GH) from the anterior pituitary gland. This regulation occurs by binding GHRH to its specific receptor, the pituitary GHRH receptor (pGHRH-R), triggering a cascade of intracellular signaling pathways. One of the key outcomes of this process is the activation of the pituitary GH/liver insulin-like growth factor-1 (IGF-1) axis, which is responsible for mediating many of the anabolic and metabolic effects attributed to GH [1–3]. IGF-1, in particular, plays a critical role in cellular growth, proliferation, and tissue repair, linking GH signaling to broader metabolic and regenerative processes [4].

In addition to its classical endocrine functions, GHRH has been detected in extrahypothalamic tissues, including the placenta, ovaries, testes, gastrointestinal tract, and even tumors [1–5]. This suggests that GHRH has broader physiological roles that extend beyond the pituitary gland. The discovery of GHRH receptors (GHRH-R) and their splice variants in various tissues and organs further supports the idea that GHRH may have direct autocrine and paracrine effects in these tissues [4–7]. When GHRH binds to its receptors, it activates multiple intracellular signaling pathways, influencing diverse biological processes such as cell proliferation, differentiation, and survival [8–10].

The human GHRH peptide, which consists of 44 amino acids, is structurally related to other members of the secretin/glucagon peptide family, including vasoactive intestinal peptide (VIP), pituitary adenylate-cyclase-activating polypeptide (PACAP), and glucagon-like peptides (GLPs) [11, 12]. This structural homology underlies some of the shared biological functions and receptor cross-reactivity observed among these peptides.

In the early 1990s, research into the potential role of GHRH in carcinogenesis spurred efforts to develop synthetic analogs of GHRH that could be used as therapeutic agents. These analogs fell into two broad categories: agonists and antagonists. Antagonistic GHRH analogs were initially designed to block the effects of GHRH on tumor cells, with the hope of exploiting the presence of GHRH receptors in certain cancer types [8]. At the same time, agonistic analogs were developed to evaluate their potential clinical applications, particularly in conditions where GH deficiency played a critical role [13–15]With interest in the possible roles of GHRH in carcinogenesis, our research group began synthesizing antagonistic analogs of GHRH to evaluate the use of cancer therapy. We also started a systematic synthesis of agonistic GHRH analogs. Here, we describe the GHRH analogs developed in our laboratory during the past decades and evaluate these peptides from a clinical perspective.

## The development of growth hormone-releasing hormone (GHRH) analogs

### Synthesis of GHRH analogs with agonistic activities

Given that the biological activity of GHRH is confined to its N-terminal sequence of 29 amino acids, most of these synthetic analogs were based on modifications of the GHRH (1–29) NH2 sequence [16, 17]The sequence of GHRH (1–29) NH2 was used to substitute and replace different amino acid residues to make agonists and antagonists of GHRH in the solid-phase peptide syntheses.

The native GHRH (1–29) NH2 peptide has a short half-life, primarily due to rapid degradation by proteolytic enzymes in circulation [2, 4]. To overcome this limitation, researchers in our laboratory focused on synthesizing degradation-resistant GHRH agonists that would retain their biological activity while exhibiting improved stability. These efforts were successful, leading to the development of analogs of the MZ-, JI-, and MR- series, that could stimulate GH secretion more effectively both *in vitro* and *in vivo* [4, 13–15] (Table 1).

**Table 1:** Chemical structure of hGHRH (1–29) agonists

	1	2	3	4	5	6	7	8	9	10	11	12	13	14	15	16	17	18	19	20	21	22	23	24	25	26	27	28	29	30
hGHRH(1–29)	**Tyr**	Ala	Asp	Ala	Ile	Phe	Thr	Asn	Ser	**Tyr**	Arg	**Lys**	Val	Leu	Gly	Gln	Leu	Ser	Ala	Arg	**Lys**	Leu	Leu	Gln	Asp	Ile	**Met**	**Ser**	**Arg**	NH2
Nle27-GHRH(1–29)	**-**	-	-	-	-	-	-	-	-	**-**	-	**-**	-	-	-	-	-	-	-	-	-	-	-	-	-	-	**Nle**	-	**-**	-
MZ-2–51	**Dat**	-	-	-	-	-	-	-	-	**-**	-	**-**	-	-	**Ala**	-	-	-	-	-	-	-	-	-	-	-	**Nle**	-	**Agm**	
MZ-2–57	**Dat**	-	-	-	-	-	-	-	-	**-**	-	**D-Lys**	-	-	**Ala**	-	-	-	-	-	-	-	-	-	-	-	**Nle**	**-**	**Agm**	
MZ-2–159	**Dat**	-	-	-	-	-	-	-	-	**D-Tyr**	-	**-**	-	-	**Ala**	-	-	-	-	-	-	-	-	-	-	-	**Nle**	**-**	**Agm**	
MZ-3–149	**Dat**	-	-	-	-	-	-	-	-	**-**	-	**-**	-	-	**Ala**	-	-	-	-	-	-	-	-	-	-	-	**Nle**	**Asp**	**Agm**	
JI-34	**Dat**	-	-	-	-	-	-	-	-	-	-	**Orn**	-	-	**Abu**	-	-	-	-	-	**Orn**	-	-	-	-	-	**Nle**	**Asp**	**Agm**	
JI-36	**Dat**	-	-	-	-	-	-	**Thr**	-	-	-	**Orn**	-	-	**Abu**	-	-	-	-	-	**Orn**	-	-	-	-	-	**Nle**	**Asp**	**Agm**	
JI-38	**Dat**	-	-	-	-	-	-	**Gln**	-	-	-	**Orn**	-	-	**Abu**	-	-	-	-	-	**Orn**	-	-	-	-	-	**Nle**	**Asp**	**Agm**	
MR-356	**N-Me-Tyr**	**-**	-	-	-	-	-	**Gln**	-	-	-	**Orn**	-	-	**Abu**	-	-	-	-	-	**Orn**	-	-	-	-	-	**Nle**	**Asp**	**Agm**	
MR-403	**N-Me-Tyr**	**D-Ala**	-	-	-	-	-	**Gln**	-	-	-	**Orn**	-	-	**Abu**	-	-	-	-	-	**Orn**	-	-	-	-	-	**Nle**	**Asp**	**-**	**-NH-CH3**
MR-409	**N-Me-Tyr**	**D-Ala**	-	-	-	-	-	-	-	-	-	**Orn**	-	-	**Abu**	-	-	-	-	-	**Orn**	-	-	-	-	-	**Nle**	**Asp**	**-**	

Comparative structure of hGHRH(1–29) NH2 and GHRH agonists of MZ-, JI- and MR- series. Amino acid residues are identical to those of hGHRH(1–29)NH2 are denoted by "-". Noncoded amino acids are abbreviated as follows: *Abu* alpha-aminobutyric acid, *Agm* agmatine, *Nle* norleucine, *N-Me-Tyr* N-methyl-tyrosine, *Orn* ornithine

[Nle^27^] GHRH(1–29)-NH_2_, the first synthetic amidated 29 amino acid peptides with the replacement of Met^27^ with Nle at position 27, was reported to stimulate GH secretion when given by iv or subcutaneous administration without adverse effects [18, 19]. Our laboratory began a systematic synthesis of analogs of GHRH with agonistic activities, using [Nle^27^] GHRH(1–29)-NH_2_ as the backbone sequence. A major focus of these efforts was to develop analogs with increased resistance to degradation by proteolytic enzymes. This was achieved through modifications at the peptide's N- and C-terminal ends. In the MZ series of GHRH agonists, for example, tyrosine at the N-terminus was replaced with 2,4-Diaminobutyric acid (Dat), while the C-terminal arginine-NH2 was replaced with agmatine [20]. These modifications significantly increased the resistance of the peptides to enzymatic degradation, while enhancing their GH-releasing potency *in vivo* [13, 20]. Additional substitutions, such as replacing glycine at position 15 with alanine or introducing D-amino acids at positions 10 and 12, further enhanced the bioactivity of these analogs [20].

One of the more notable peptides in the MZ series, MZ-3–149, contained an aspartic acid residue at position 28, which allowed for efficient delivery via pulmonary inhalation—a significant advancement in the method of administration [21]. This peptide demonstrated a strong stimulatory effect on GH release *in vivo*, highlighting the potential of inhaled GHRH agonists for clinical use.

In the JI series of analogs, the focus was on reducing degradation by trypsin and other proteolytic enzymes. This was achieved by replacing lysine at positions 12 and 21 with ornithine (Orn), and alanine at position 15 with α-aminobutanoyl (Abu) [14] (Table 1). These analogs were extensively tested *in vivo*, and several, including JI-34, JI-36, and JI-38, displayed GH-releasing potency that was 89.7, 87.8, and 116.8 times greater than that of native GHRH (1–29) NH2, respectively [14, 21]. These analogs also exhibited higher binding affinities for GHRH receptors on rat pituitary cells, making them strong candidates for further therapeutic evaluation.

### Therapeutic applications of GHRH agonists

The GHRH agonists developed during this period showed promise not only in their ability to stimulate GH secretion but also in their effects on a variety of non-endocrine tissues. For instance, JI-34 was found to protect human lung microvascular endothelial cells (HL-MVECs) from pulmonary permeability edema induced by pneumolysin [22, 23]. This protection was attributed to the ability of JI-34 to bind to GHRH receptors expressed on endothelial cells, thereby triggering intracellular signaling pathways that promote cell survival.

JI-34 also demonstrated efficacy in promoting the viability and mobility of mesenchymal stem cells (MSCs), leading to significant improvements in therapeutic angiogenesis in a mouse model of critical limb ischemia [24]. The ability of GHRH agonists to enhance MSC survival and function has implications for regenerative medicine, particularly in the context of tissue repair and vascular regeneration.

The potential therapeutic applications of GHRH agonists extended to diabetes as well. GHRH receptors were found to be expressed in both human and rat pancreatic islets, where they appeared to play a role in β-cell survival and proliferation [25, 26]. JI-36, in particular, was shown to increase the size of islets and promote glucose-responsive insulin secretion in isolated rat islets. Pretreatment of islets with JI-36 before transplantation into diabetic mice significantly enhanced engraftment and improved metabolic function [25]. These findings suggest that GHRH agonists could have therapeutic potential in diabetes, particularly in enhancing the success of islet transplantation for type 1 diabetes (T1D).

The cardioprotective effects of GHRH agonists were another area of interest. GHRH receptors were detected in cardiomyocytes, suggesting a potential role for GHRH signaling in the heart [27]. JI-38, for example, was shown to stimulate cardiomyocyte survival and promote myocardial repair after experimental myocardial infarction in rats [27, 28]. This analog also accelerated wound healing by stimulating fibroblast proliferation and promoting the reformation of the epithelium at later stages of healing [29, 30]. These findings suggest that GHRH agonists could have broad applications in tissue repair and regeneration, particularly in cardiovascular and wound healing contexts.

### MR series of GHRH agonists

Building on the success of the JI series, researchers developed the MR series of GHRH agonists, which were designed to have even higher biological potency and improved pharmaceutical properties [15]. Nearly 100 peptides were synthesized in this series, with modifications including the incorporation of N-terminal N-methyl-tyrosine (N-Me-Tyr) and C-terminal methyl- or ethyl-amides. replacement of Ala^2^ with D-Ala [16] and substitution of amino acids at different positions. Key substitutions from the JI series, such as replacing lysine at positions 12 and 21 with ornithine (Orn) and alanine at position 15 with α-aminobutanoyl (Abu), were retained in many of the MR series analogs [15].

Among the MR series, MR-356, MR-403, and MR-409 were particularly notable for their endocrine activity. These analogs demonstrated several-fold higher GH-releasing potency than JI-38 upon subcutaneous administration *in vivo* and exhibited greater binding activity to GHRH receptors *in vitro* [15]. MR-409 emerged as a strong candidate for further therapeutic development due to its wide range of biological activities in various animal models [2, 15].

In rodent and swine models of myocardial infarction, MR-356, and MR-409 demonstrated potent cardioprotective effects, including attenuation of cardiac hypertrophy and improved heart function [31–37]. This analog also reduced vascular calcification in osteoprotegerin-deficient mice and improved cardiac function in aged mice [38, 39], and ameliorated disc degeneration in rats [40]. Additionally, MR-409 exhibited neuroprotective effects in models of spinal muscular atrophy (SMA), where it promoted weight gain, improved motor behavior, and enhanced neuromuscular junction maturation [41]. In models of ischemic stroke, MR-409 facilitated endogenous neurogenesis and neuroplasticity, leading to improved functional recovery [42]. Furthermore, in a rat model of induced optic nerve injury, subcutaneous administration of MR-409 reduced the degeneration of retinal ganglion cells (RGCs) and enhanced the neuroprotective effects of macrophage activation on RGC survival [43]. Interestingly, MR-409 also promoted wound healing by stimulating the proliferation and survival of human dermal fibroblasts. The topical application of MR-409 accelerated the healing of skin wounds in mice [44].

MR-409 demonstrated significant anti-inflammatory properties, as evidenced by its ability to attenuate dextran sodium sulfate-induced colitis in mice [45]. Moreover, the analog exhibited antioxidant effects and induced anxiolytic and antidepressant-like behavior *in vivo* [46]. These findings suggest that MR-409 has potential applications in a wide range of inflammatory and neurodegenerative conditions.

### GHRH agonists in diabetes

Evaluation of the potential of the MR series of agonists on the survival and metabolic functions of rat pancreatic β-cells revealed that MR-403, MR-356, and MR-409 significantly increased β-cell viability and proliferation while reducing apoptosis [2, 47]. Further studies showed that MR-409 reduced the severity of diabetes in a streptozotocin-induced mouse model. Transplantation of rat islets preconditioned *in vitro* with MR-409, along with *in vivo* administration, promoted the growth, function, and engraftment of exogenous islets [47]. Additional research demonstrated the survival mechanisms of MR-409 in human and rodent β-cells, as well as in preclinical models of type 1 diabetes (T1D) [48].

The finding that MR-409 induced the expression of insulin receptor substrate 2 (IRS2), a master regulator of β-cell survival and growth, suggests the potential benefits of the GHRH agonist in type 2 diabetes (T2D) [48]. In a study with diabetic db/db mice, MR-409 treatment lowered plasma lipid levels, attenuated vascular calcification, and reduced heart valve calcification, though it did not significantly affect blood glucose levels or insulin tolerance [49].

### GHRH agonists in cancer therapy

Despite initial concerns that GHRH agonists might stimulate tumor growth, studies have shown that these compounds may, in fact, inhibit the proliferation of certain cancer types. *In vitro* studies using human lung cancer cell lines, including small-cell lung cancer (SCLC) H446 and non-small-cell lung cancer (NSCLC) HCC827 and H460 cells, demonstrated that GHRH agonist MR-409 exerted significant agonistic activity, inhibiting tumor growth [50]. The *in vivo* studies in nude mice xenografted with human lung tumors further revealed that MR-409 significantly suppressed tumor growth [50]. Likewise, treatment of nude mice bearing human gastric tumor (NCI-N87) with MR-356, significantly inhibited the growth of the tumor and, lowered serum IGF-1 levels [51]. This inhibition of tumor growth was comparable to that observed with the GHRH antagonist MIA-602, suggesting that the agonist paradoxically downregulated GHRH receptors in the tumor microenvironment [2, 52].

Further studies showed that MR-409 reduced the growth of a variety of other human tumors when tested in xenograft models. These tumors included gastric cancer (NCI-N87), pancreatic cancer (CFPAC-1 and PANC-1), bladder cancer, prostate cancer (PC-3), triple-negative breast cancer (MDA-MB-231), and colorectal cancer (HCT-116 and HCT-15) [50]. The degree of tumor inhibition varied across cancer types, but no detectable stimulation of tumor growth was observed in any of the models [50, 51].

Proteomic studies conducted on tumor cells treated with GHRH agonists have suggested that these compounds may exert their anti-tumor effects by inducing the differentiation of cancer cells [53]. Additionally, the downregulation of pituitary and tumoral GHRH receptors by prolonged agonist administration may contribute to the inhibition of tumor growth [50, 54]. These findings are reminiscent of the downregulation of luteinizing hormone-releasing hormone (LHRH) receptors observed during therapy with LHRH agonists, which is known to suppress the growth of hormone-dependent cancers such as prostate and breast cancer [55–57].

## In Summary

The development of GHRH agonists has opened new therapeutic avenues, offering a wide range of potential applications in fields as diverse as endocrinology, cardiology, neurology, and oncology. The MR series, particularly MR-409, represents a promising candidate for translational development in multiple clinical settings. Its ability to stimulate GH release while exerting protective effects on the heart, nervous system, and immune system suggests that GHRH agonists may have broad clinical utility. Moreover, the surprising anti-tumor effects observed with MR-409 in various cancer models provide new insights into the potential pharmaceutical applications of GHRH agonists in oncology. The ability of GHRH agonists to modulate both endocrine and extra-endocrine pathways makes them versatile tools for the treatment of a variety of diseases, from metabolic disorders to cancer.

## The development of growth hormone-releasing hormone (GHRH) antagonists

### GHRH antagonists and their role in cancer therapy

Although GHRH was first identified in tumor tissues in earlier 1980s [58, 59], its potential role in carcinogenesis was not extensively explored until the mid-1990s. This oversight was primarily due to the prevailing view that GHRH was limited to its role in regulating growth hormone (GH) secretion from the pituitary gland. However, as research began to show the presence of GHRH and its receptors in various extrahypothalamic tissues, including tumor cells, the possible oncogenic or anti-oncogenic effects of GHRH came under scrutiny [8]. With our extensive experience and interest in oncology, we turned our focus toward the synthesis of GHRH antagonists and their potential application in cancer therapy [8, 9, 60–62].

By incorporating non-natural amino acids, large polymeric tags, or amide bond replacements, along with conformational constraints, we synthesized several hundred peptides grouped into different series: MZ, JV, MZ-J, JMR, MIA, and AVR [2, 60–68]. These synthetic GHRH antagonists were evaluated for their receptor-binding affinity, resistance to proteolytic degradation, and bioactivity *in vitro* and *in vivo*, in comparison with natural human GHRH (1–29) NH2 or the first described synthetic GHRH antagonist, (N-Ac-Tyr1, D-Arg2)-GHRH (1–29)NH2 [69]. In this early antagonist, the modification at the N-terminus and the replacement of Ala2 with D-Arg2 resulted in significant suppression of GH release in rats [69].

### First-generation GHRH antagonists: MZ and JV series

The MZ series was among the first group of GHRH antagonists synthesized (Table 2). In this class, hydrophobic acylation with moieties such as isobutyric acid (Ibu), phenylacetic acid (PhAc), or 1-naphthylacetic acid (Nac) replaced the acetic acid (Ac) at the N-terminus [62]. Additionally, enzyme-resistant modifications at the C-terminus included 1-amino-4-guanidinobutane (Agm, agmatine). Other modifications such as replacing Phe^6^ with 4-chloro-Phe (Cpa), Gly^15^ with α-aminobutyric acid (Abu), and Met^27^ with norleucine (Nle) further enhanced the stability and potency of these antagonists [62, 63]. Some potent antagonists from this series included MZ-4–71, MZ-4–243, and MZ-5–156, which exhibited receptor binding affinities 20–100 times greater than the standard antagonist. MZ-4–71 inhibited GH release 18.9-fold more effectively than the standard antagonist [9, 62, 63].

**Table 2:** Chemical structure of hGHRH (1–29) antagonists

	**0**	**1**	**2**	**3**	**4**	**5**	**6**	**7**	**8**	**9**	**10**	**11**	**12**	**13**	**14**	**15**	**16**	**17**	**18**	**19**	**20**	**21**	**22**	**23**	**24**	**25**	**26**	**27**	**28**	**29**	**30**
hGHRH(1–29)	**H-**	Tyr	**Ala**	Asp	Ala	Ile	**Phe**	Thr	**Asn**	**Ser**	**Tyr**	**Arg**	**Lys**	Val	Leu	**Gly**	Gln	Leu	Ser	Ala	**Arg**	**Lys**	Leu	Leu	Gln	Asp	Ile	**Met**	**Ser**	**Arg**	**NH2**
Standard	**Ac**	**-**	**D-Arg**	-	-	-	-	-	-	-	-	-	-	-	-	-	-	-	-	-	-	-	-	-	-	-	-	-	-	-	-
MZ-4–71	**Ibu**	**-**	**D-Arg**	-	-	-	**Cpa**	-	-	-	-	-	-	-	-	**Abu**	-	-	-	-	-	-	-	-	-	-	-	**Nle**	-	**Agm**	
MZ-4–243	**Nac**	-	**D-Arg**	-	-	-	**Cpa**	-	-	-	-	-	-	-	-	**Abu**	-	-	-	-	-	-	-	-	-	-	-	**Nle**	-	**Agm**	
MZ-5–156	**PhAC**	**-**	**D-Arg**	-	-	-	**Cpa**	-	-	-	-	-	-	-	-	**Abu**	-	-	-	-	-	-	-	-	-	-	-	**Nle**	-	**Agm**	
JV-1–36	**PhAC**	**-**	**D-Arg**	-	-	-	**Cpa**	-	-	**Arg**	-	-	-	-	-	**Abu**	-	-	-	-	-	-	-	-	-	-	-	**Nle**	**D-Arg**	**Har**	**-**
JV-1–38	**PhAC**	**-**	**D-Arg**	-	-	-	**Cpa**	-	-	**Har**	**Tyr(Me)**	-	-	-	-	**Abu**	-	-	-	-	-	-	-	-	-	-	-	**Nle**	**D-Arg**	**Har**	-
MZJ-7–46	**CH3-(CH2)6-CO**	**-**	**D-Arg**	-	-	-	**Cpa**	-	-	**Arg**	**-**	-	-	-	-	**Abu**	-	-	-	-	-	-	-	-	-	-	-	**Nle**	**D-Arg**	**Har**	-
JMR-132	**PhAC**	**-**	**D-Arg**	-	-	-	**Cpa**	-	**Ala**	**Har**	**Tyr(Me)**	**His**	**-**	-	-	**Abu**	-	-	-	-	**His**	-	-	-	-	-	-	**Nle**	**D-Arg**	**Har**	-
MIA-602	**PhAC-Ada**	-	**D-Arg**	-	-	-	**5FPhe**	-	**Ala**	**Har**	**Tyr(Me)**	**His**	**Orn**	-	-	**Abu**	-	-	-	-	**His**	**Orn**	-	-	-	-	-	**Nle**	**D-Arg**	**Har**	-
MIA-690	**PhAC-Ada**	**-**	**D-Arg**	-	-	-	**Cpa**	-	**Ala**	**Har**	**5FPhe**	**His**	**Orn**	-	-	**Abu**	-	-	-	-	**His**	**Orn**	-	-	-	-	-	**Nle**	**D-Arg**	**Har**	-
AVR-352	**5FPhAC-Ada**	**-**	**D-Arg**	-	-	-	**5FPhe**	-	**Ala**	**Har**	**Tyr(Me)**	**-**	**-**	-	-	**Abu**	-	-	-	-	**-**	**-**	-	-	-	-	-	**Nle**	**D-Arg**	**Har**	**Ada-NH2**
AVR-353	**5FPhAC-Ada**	**-**	**D-Arg**	-	-	-	**Cpa**	-	**Ala**	**Har**	**5FPhe**	**-**	**-**	-	-	**Abu**	-	-	-	-	**-**	**-**	-	-	-	-	-	**Nle**	**D-Arg**	**Har**	**Ada-NH3**

Comparative structure of hGHRH(1–29) NH2 and GHRH antagonists of MZ-, JV-1-, MZJ-, JMR-, MIA-, and AVR- series. Amino acid residues are identical to those of hGHRH(1–29)NH2 are denoted by "-". Noncoded amino acid are abbreviated as follows: 5FPhe pantafluoro-phynylalanine, 5FPh AC- pentafluoro-phenylacetyl, *Abu* alpha-aminobutanoyl, *Ac* acetyl, *Ada* 12-aminododecanoyl, *Agm* agmatine, *Cpa* 4-chloro-phenyalanine, *Har* Homoarginine, *Ibu* isobutyric acid, *Nac* 1-naphthylacetic acid, *Nle* norleucine, *N-Me-Tyr* N-methyl-tyrosine, *Orn* ornithine, *PhAc* phenylacetic acid, *Tyr(Me)* O-methyl-tyrosine

Following the success of the MZ series, the JV series introduced further refinements, including the substitution of Ser^9^ with hydrophilic amino acids such as Arg or homoarginine (Har), and Tyr^10^ with Tyr(Me). The C-terminal agmatine was replaced with enzymatically resistant sequences such as D-Arg^28^-Har. Within this series, JV-1–36 showed the highest GHRH antagonistic activity and enhanced receptor binding affinity [64]. Both JV-1–36 and JV-1–38 induced strong and prolonged inhibition of GH release *in vivo* [64]. These findings laid the groundwork for further modifications to enhance receptor affinity and antitumor activity while reducing GH release suppression.

### Development of MZ-J, JMR, and MIA series of antagonists

Building on the success of the JV series, we developed the MZ-J series by further modifying JV-1–36 with octanoic acid at the N-terminus, resulting in analog MZ-J-7–46, which exhibited greater antitumor activity while maintaining high receptor affinity [65, 66]. This modification helped improve the pharmacokinetic properties of the antagonist, making it more resistant to enzymatic degradation *in vivo*.

The JMR series continued this trend by modifying JV-1–38. In this series, Arg at positions 11 and 20 was replaced with His, and Asn^8^ was replaced with Ala. These changes resulted in even higher receptor binding affinity and greater antitumor effects *in vitro* and *in vivo* [9, 60]. Despite their enhanced antitumor effects, these antagonists had relatively weaker inhibitory effects on GH release compared to earlier analogs [9, 60].

Studies evaluating the antitumor activities of the MZ, JV, MZ-J, and JMR series were conducted on various human cancer cell lines *in vitro*, as well as in nude mice bearing xenografted human tumors [8, 9, 60–62]. These antagonists effectively inhibited the growth of several types of human cancers, including osteogenic sarcomas (SK-EF-1, MNNG/HOS) [70, 71], small-cell lung cancer (SCLC, H69, H460, DMS-153), non-small cell lung cancer (NSCLC, H157, A-549) [72–76], renal adenocarcinoma (Caki-I) [77], androgen-independent prostate cancer (DU-145, PC-3) [78–81], estrogen-independent breast cancer (MDA-MB-468, MXT) [82, 83], endometrial cancer [84], pancreatic carcinoma (SW1990) [85], colorectal cancer (HT-29) [86], U-87 MG glioblastoma [86], and non-Hodgkin’s lymphoma (RL and HT) [87].

In these studies, multiple mechanisms of action were identified for GHRH antagonists. They exerted their effects by binding to GHRH receptors on cancer cells, blocking the signaling pathways that promote tumor growth and survival. These antagonists also reduced the expression of various growth factors and pro-inflammatory cytokines associated with tumor progression [60].

### MIA series: Enhancing stability and antitumor activity

We synthesized the MIA series of antagonists to develop GHRH antagonists with increased stability, longer half-life, and stronger antitumor activity [67]. In this series, several key features of JMR-132, such as D-Arg^2^, Ala^8^, Har^9^, His^11^, Abu^15^, His^20^, Nle^27^, D-Arg^28^, and Har^29^, were maintained, while additional modifications focused on increasing resistance to proteolytic degradation. This was achieved by incorporating a long hydrocarbon chain (Ada) at the N-terminus and substituting Phe at position 6 or Tyr at position 10 with pentafluorophenylalanine (5FPhe), as fluorine substitution has been shown to improve metabolic stability [67]. Ornithine (Orn) was replaced with lysine (Lys) at positions 12 and 21 to reduce degradation by trypsin [67].

Among the 22 synthesized analogs in the MIA group, MIA-602 emerged as one of the most promising compounds, with higher receptor binding affinities and strong inhibitory effects on cancer cell proliferation *in vitro* and tumor growth *in vivo*. MIA-602 demonstrated remarkable antitumor activity across various cancer models. The complete sequences of MIA-602 were shown in Table 2, MIA-602 composed modification at position 0. 2, 6. 8, 9 10, 11, 12, 15, 20, 21, 27, 28, 29 of GHRH (1–29)-NH2 [67]. However, like JMR-132, MIA 602 had a weak inhibitory effect on GH release *in vivo*.

### AVR series: Improved antitumor and anti-inflammatory potency

The AVR series of GHRH antagonists represents the latest advancement in the development of these compounds. The AVR series builds upon the structure of MIA-602 and MIA-690 by modifying the N-terminus with pentafluoroPhAc-Ada and the C-terminus with Har29-Ada-NH2 [68]. These modifications further enhanced the receptor-binding affinity of AVR compounds, with some analogs exhibiting 2- to 4.5-fold higher affinity compared to MIA-602 [68]. Among these, AVR-352 and AVR-353 demonstrated greater inhibitory potency against the growth of human cancers, including lung cancer (HCC827, H460), pancreatic cancer (CFPAC-1, PANC-1), gastric cancer (NCI N87), colorectal cancer (HT-29), breast cancer (MX-1), ovarian cancer (SK-OV-3, OVCAR-3), prostate cancer (PC-3), and glioblastoma (U87) [68].

In addition to their antitumor effects, AVR-352 and AVR-353 also showed enhanced suppressive activity on GH release *in vivo* compared to MIA-602. AVR-352 exhibited strong anti-inflammatory effects in a mouse model of lung inflammation, suggesting that these antagonists may have applications beyond oncology, particularly in inflammatory diseases [68].

### Mechanisms of action and antitumor effects of GHRH antagonists

Extensive research has demonstrated that GHRH antagonists exert their antitumor effects by binding to GHRH receptors expressed on tumor cells, thereby blocking GHRH-mediated signaling pathways involved in tumor growth, invasion, and survival [8, 9, 60, 61]. In addition to directly inhibiting tumor cell proliferation, GHRH antagonists reduce the secretion of growth factors such as IGF-1, VEGF, and pro-inflammatory cytokines, which are essential for tumor angiogenesis and metastasis [8, 9, 60, 61].

Among the antagonists synthesized in our laboratory, the biological activities of MIA-602 were extensively studied in various experimental cancer models *in vitro* and *in vivo*. In addition to the types of cancers studied with antagonists of MZ-, JV-1-, and JMR- series, such as endometrial cancer (Hec-1) [67, 88, 89], ACHN renal carcinoma [67], SCLC (H460) [52], U-87 glioblastoma [90], breast [91, 92], colon [93]. MIA-602 showed strong inhibition on tumor growth in other types of cancer models such as esophageal squamous cell carcinoma [94], gastric cancer [95], pleural mesothelioma [96], androgen-independent, androgen-dependent, and castrate-resistant prostate [97, 98], pituitary adenomas [99], melanoma [100], acute myeloid leukemia [101], acute promyelocytic leukemia [102], and Thyroid cancer [103, 104]. When used in conjunction with other treatments, the synergistic effects highlight the potential of GHRH antagonists as adjuvant therapies in cancer treatment [92, 105].

### Anti-inflammatory effects of GHRH antagonists

Beyond their antitumor activities, GHRH antagonists have demonstrated potent anti-inflammatory effects in various disease models. In animal studies, GHRH antagonists such as JMR-132 and MIA-609 significantly reduced prostate weight and volume in models of benign prostatic hyperplasia and autoimmune prostatitis, primarily by suppressing pro-inflammatory cytokines and altering epithelial-to-mesenchymal transition (EMT) pathways [106–108].

Moreover, MIA-602 was found to reduce retinal ganglion cell degeneration in rats subjected to optic nerve injury and alleviate experimental ocular inflammation [2, 43]. GHRH antagonists were shown to enhance pulmonary endothelial barrier integrity [109].

In lung injury models, MIA-602 reduced inflammation and fibrosis by modulating the immune response, offering potential applications in treating lung diseases such as pulmonary fibrosis [110, 111], and sarcoidosis-like granuloma [111]. Recent studies also demonstrated that MIA-602 inhibited pro-inflammatory gene expression in alveolar epithelial cells (iAT2), implicating GHRH-R signaling in lung inflammation and fibrosis [112]. Additionally, MIA-602 reduced inflammation in THP-1-derived macrophages and PBMCs stimulated with the SARS-CoV-2 spike protein [113].

Notably, MIA-602 showed protective effects in a BSL-2 mouse model of SARS-CoV-2-induced pulmonary dysfunction and heart injury [114]. Treatment with MIA-602 reduced airway inflammation improved respiratory parameters, and restored normal airflow, suggesting that GHRH antagonists may have therapeutic potential in viral infections and associated complications [114].

### Neuroprotective effects of GHRH antagonists

Chronic treatment with MIA-602 and MIA-690 has demonstrated anxiolytic and antidepressant-like effects in animal models, likely through reducing inflammation and oxidative stress while promoting synaptogenesis [46, 115]. MIA-690 also exhibited beneficial effects in inhibiting amyloid aggregation and proteotoxicity in a transgenic mouse model of Alzheimer’s disease, providing evidence for the neuroprotective potential of GHRH antagonists in neurodegenerative disorders [116–118].

## Conclusion

The development of GHRH agonists focused on enhancing GH release, improving metabolic functions, and promoting tissue repair. MR-409 demonstrated therapeutic potential across various conditions, from cardioprotection to neuroprotection. The development of GHRH antagonists, particularly those in the MIA and AVR series, has provided new opportunities for cancer therapy and the treatment of other diseases. These antagonists have shown efficacy in antitumor and anti-inflammatory activities with minimal adverse effects, positioning them as favorable alternatives to conventional chemotherapeutic agents. MIA-602 and AVR-352 offer promising clinical applications for human cancers, inflammation-related diseases, and neurodegenerative disorders. The ongoing research and development of GHRH agonists and antagonists continue to highlight their potential as versatile therapeutic agents across a wide range of medical conditions.

## Data Availability

No datasets were generated or analysed during the current study.
